# In Vitro Inhibitory Potential of Different Anthocyanin-Rich Berry Extracts in Murine CT26 Colon Cancer Cells

**DOI:** 10.3390/molecules28237684

**Published:** 2023-11-21

**Authors:** Cornelia Schmutz, Frank Will, Elisabeth Varga, Carola Jaunecker, Gudrun Pahlke, Walter Berger, Doris Marko

**Affiliations:** 1 Department of Food Chemistry and Toxicology, Faculty of Chemistry, University of Vienna, Währingerstraße 38-40, 1090 Vienna, Austria; cornelia.schmutz@univie.ac.at (C.S.); elisabeth.varga@vetmeduni.ac.at (E.V.); gudrun.pahlke@univie.ac.at (G.P.); 2 Doctoral School in Chemistry, University of Vienna, Währingerstraße 42, 1090 Vienna, Austria; 3 Department of Beverage Research, Hochschule Geisenheim University, P.O. Box 1154, 65366 Geisenheim, Germany; frank.will@hs-gm.de; 4 Center for Cancer Research and Comprehensive Cancer Center, Medical University of Vienna, Borschkegasse 8a, 1090 Vienna, Austria; carola.jaunecker@meduniwien.ac.at (C.J.); walter.berger@meduniwien.ac.at (W.B.)

**Keywords:** adhesion, cytotoxicity, DNA damage, flavonoids, irinotecan, polyphenols

## Abstract

Anti-oxidant, -inflammatory, and -carcinogenic activities of bioactive plant constituents, such as anthocyanins, have been widely discussed in literature. However, the potential interaction of anthocyanin-rich extracts with routinely used chemotherapeutics is still not fully elucidated. In the present study, anthocyanin-rich polyphenol extracts of blackberry (BB), bilberry (Bil), black currant (BC), elderberry (EB), and their respective main anthocyanins (cyanidin-3-*O*-glucoside, delphinidin-3-*O*-glucoside, cyanidin-3-*O*-rutinoside, and cyanidin-3-*O*-sambubioside) were investigated concerning their cytotoxic and DNA-damaging properties in murine CT26 cells either alone or in combination with the chemotherapeutic agent SN-38. BB exerted potent cytotoxic effects, while Bil, BC, and EB only had marginal effects on cell viability. Single anthocyanins comprised of the extracts could not induce comparable effects. Even though the BB extract further pronounced SN-38-induced cytotoxicity and inhibited cell adhesion at 100–200 µg/mL, no effect on DNA damage was observed. In conclusion, anti-carcinogenic properties of the extracts on CT26 cells could be ranked BB >> BC ≥ Bil ≈ EB. Mechanisms underlying the potent cytotoxic effects are still to be elucidated since the induction of DNA damage does not play a role.

## 1. Introduction

Anthocyanins are ubiquitously occurring secondary plant metabolites responsible for the vibrant colour range of red over blue to purple in numerous plants, fruits, and vegetables [1]. Due to their anti-oxidative and anti-inflammatory properties, anthocyanins are reported to be promising substances regarding chemoprevention [2,3,4]. Furthermore, the anti-carcinogenic properties of anthocyanins and anthocyanin-rich extracts have been studied intensely over the last decades, hinting towards their capacity to interfere with cancer initiation, promotion and progression. Considering that less than 1.8% of anthocyanins are absorbed from the gastrointestinal tract (GIT) and, hence, systemically bioavailable, local interactions with epithelial cells of the GIT are of specific interest [5]. For instance, several recent studies focused on the anti-proliferative effect of anthocyanins on colorectal cancer (CRC) [6,7,8,9]. It has been reported that—depending on the sugar moiety—28–85% of intact anthocyanins from blueberries reach the ileostomy bags of patients and could therefore reach the colon [10]. In vivo studies showed that diets containing 10% freeze-dried black raspberries reduced chemically induced tumor incidence not only in the esophagus of N344 rats but also in their colon [11]. Furthermore, feed containing 10% bilberry extract protected mice against reduction of colon length and increase in tumor numbers induced by dextran sodium sulfate and azoxymethane, respectively [12].

Chemotherapeutic treatments for CRC, which is the third-most frequent malignancy leading to cancer-related deaths, routinely consist of combinations of 5-fluoruracil (5-FU) and leucovorin, 5-FU, and irinotecan (CPT-11) or CPT-11 alone [13,14,15,16]. SN-38, the toxic metabolite of CPT-11, is released in the body by carboxyl esterase activity and acts as a potent topoisomerase (topo) I poison. SN-38 hence stabilizes the covalent DNA/topo I intermediate, thereby trapping the intermediate, leading to collisions with the replication fork and ultimately to persistent DNA strand breaks and apoptosis. Anthocyanins have also been reported to interact with topos as catalytic inhibitors, which in high concentrations might also impair DNA integrity due to suppression of torsion stress control. For instance, the anthocyanidins cyanidin and delphinidin not only inhibited topos I and II activity in cell free assays but also induced DNA damage at concentrations ≥50 µM in HT29 cells [17].

As cell adhesion is part of a myriad of cell regulation processes, changes can be crucial incidents in the development of several diseases, including atherosclerosis, arthritis, osteoporosis, and cancer [18]. The interaction of anthocyanins with cell adhesion molecules such as ICAM-1 (intercellular adhesion molecule 1) and VCAM-1 (vascular cell adhesion molecule 1) has hence been the subject of several studies, hinting towards their ability to reduce cell adhesion and therefore potentially interfere with carcinogenesis [19,20].

In the present study, anthocyanin-rich extracts of the four berries, blackberry (*Rubus fructicosus*, BB), bilberry (*Vaccinium myrtillus*, Bil), black currant (*Ribes nigrum*, BC), and elderberry (*Sambucus nigra*, EB), were produced from natural berry juices and analyzed with respect to their anthocyanin profiles. Chemical structures of selected anthocyanins cyanidin-3-*O*-glucoside (Cy3glc), delphinidin-3-*O*-glucoside (Del3glc), cyanidin-3-*O*-rutinoside (Cy3rut), and cyanidin-3-*O*-sambubioside (Cy3sam) are depicted in Supplementary Figure S1. The impact of these berry extracts as well as selected, highly abundant single anthocyanins in the context of cytotoxicity, genotoxicity, and cell adhesion on murine CT26 colon cancer cells was investigated. The aim was not only to compare the inhibitory potential of the different berry extracts alone but also in combination with the topo I poison SN-38. In anticipation of potential further in vivo studies, cells suitable for a murine allograft model were chosen. This study aims to elucidate differences in anti-carcinogenic berry extract activity and potential interactions with established chemotherapeutic treatment on murine CRC cells.

## 2. Results

### 2.1. Anthocyanin Content of Berry Extracts

High-performance liquid chromatography (HPLC) analysis and liquid chromatography mass spectrometry (LC-MS) assignation of the four berry extracts revealed a high diversity in their anthocyanin profile (Table 1, Figure S2). Due to the identical molecular masses of the hexoses glucose and galactose, differentiation of their mass spectra is not possible. However, their retention times differ in the herein presented chromatographic conditions and peaks could hence be safely assigned to the corresponding substances (Figure S2 and Table S1). The Bil extract comprised the highest amount of anthocyanins, with roughly 103 g/kg, followed by the EB, BB, and BC extracts, with 78, 76, and 46 g/kg, respectively. Furthermore, in the Bil extract, glycosides of all five major anthocyanidins could be detected, totaling 14 different anthocyanins. Glucosides represent the most prevalent anthocyanins in the Bil extract, with Del3glc and Cy3glc at 16 and 13%, respectively, as the main components. Four of the six detected anthocyanins in the BC extract were rutinosides. Delphinidin-3-*O*-rutinoside (Del3rut, 41%) and Cy3rut (39%) represent the main proportion in BC extract, while the BB extract mainly consists of Cy3glc (89%) and only 3% of Cy3rut. Other detected anthocyanins in the BB extract are cyanindin-3-*O*-xyloside (Cy3xyl) and two side-chain-decorated glucosides, namely cyanidin-3-*O*-(6″-*O*-dioxalyl)-glucoside (Cy3dioxglc, 5%) and cyanidin-3-*O*-(6″-*O*-malonyl)-glucoside (Cy3malglc, 2%). The simplest anthocyanin profile was found in the EB extract, which consists of 73% Cy3sam and 27% cyanidin-3-*O*-sambubioside-5-*O*-glucoside (Cy3sam5glc).

### 2.2. Cytotoxic Effects of Berry Extracts and Single Anthocyanins

**Figure 1:**
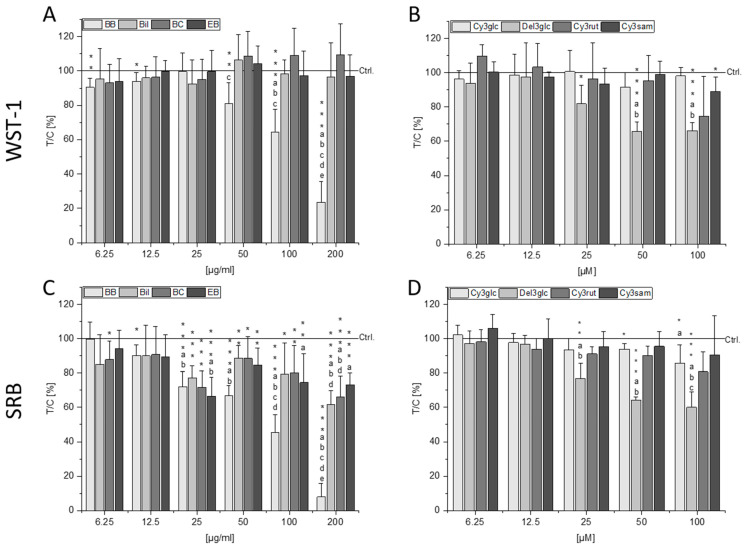
Cytotoxic properties of berry extracts of blackberry (BB), bilberry (Bil), black currant (BC) and elderberry (EB) (**A**,**C**) and single anthocyanins cyanidin-3-*O*-glucoside (Cy3glc), delphinidin-3-*O*-glucoside (Del3glc), cyanidin-3-*O*-rutinoside (Cy3rut), and cyanidin-3-*O*-sambubioside (Cy3sam) (**B**,**D**) assessed with the water-soluble tetrazolium (WST-1, **top**) and sulforhodamine B (SRB, **bottom**) assay. CT26 cells were incubated for 72 h with the respective substances. Results presented are the means + SD of at least three independent biological replicates measured in technical triplicates relative to the solvent control (Ctrl., 0.6% DMSO). Statistical differences to the control were calculated with one-sample Student’s *t*-tests (* *p* < 0.05, ** *p* < 0.01, *** *p* < 0.001) and among the concentrations with one-way ANOVA via post hoc Bonferroni test (*p* < 0.05, a–e; e.g., the letter a represents significance compared to the lowest concentration of 6.25 µg/mL or µM).

Potential cytotoxic effects of the BB, Bil, BC, and EB extracts as well as their main single anthocyanins—Cy3glc, Del3glc, Cy3rut, and Cy3sam, respectively—were examined with the coupled water-soluble tetrazolium (WST-1) and sulforhodamine B (SRB) assay. An exposure of 72 h was chosen to facilitate interpretation of potential interaction with long-term therapeutic effects in combination with the drug SN-38 in Section 2.3. Potent, concentration-dependent cytotoxic effects were observed with BB from concentrations of 50 µg/mL and 12.5 µg/mL in the WST-1 and SRB assays, respectively (Figure 1A,C). At the highest tested concentration (200 µg/mL), reductions in CT26 cell viability to 23.5 ± 12% in the WST-1 and to 7.9 ± 8% in the SRB assay (*p* < 0.001) were observed. Bil, BC, and EB extracts did not cause significant reduction in the WST-1 assay compared to the control (Figure 1A). However, in the SRB assay, a reduction in cell protein content was observed for all three extracts, starting from a concentration of 25 µg/mL with comparable potency (Figure 1C), indicating potential metabolic stress mediated by these extracts observed as higher signals in the WST-1 assay.

The single anthocyanins Cy3glc, Cy3rut, and Cy3sam could only marginally affect cell viability in both assays, if at all (Figure 1B,D). Cy3glc, the main anthocyanin of the BB extract, significantly reduced cell protein content at 50–100 µM (*p* < 0.05) but failed to do so in concentrations that are contained in the BB extract (200 µg/mL BB comprise 29.9 µM Cy3glc). Only Del3glc mediated a significant, concentration-dependent reduction in cell viability starting from a concentration of 25 µM in both the WST-1 and SRB assays. However, this concentration also exceeds what can be found in the highest concentration of Bil extract used as 200 µg/mL Bil would only contain 7.4 µM Del3glc.

### 2.3. Combinatory Cytotoxic Effects

**Figure 2:**
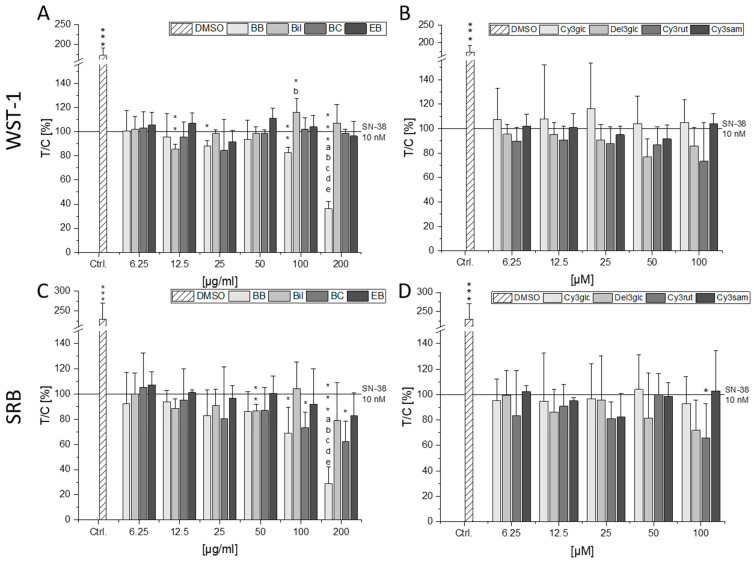
Combined cytotoxic effects of anthocyanin-rich extracts (**A**,**C**) or single anthocyanins (**B**,**D**) and SN-38. CT26 cells were pre-incubated for 30 min with anthocyanin and subsequently additionally challenged with 10 nM SN-38 for 72 h. Metabolic cell activity and cell protein content were measured with WST-1 (**A**,**B**) and SRB (**C**,**D**) assays. Data presented are the means + SD of at least three independent replicates measured in technical triplicates and expressed as test over control (T/C) in percent relative to 10 nM SN-38. Statistical significances compared to the SN-38 incubation were calculated with one-sample Student’s *t*-tests (* *p* < 0.05, ** *p* < 0.01, *** *p* < 0.001). Significances among the tested concentrations were calculated with one-way ANOVA via post hoc Bonferroni test (*p* < 0.05, a–e; e.g., the letter a represents significance compared to the lowest concentration of 6.25 µg/mL or µM).

To investigate the impact of the berry extracts and single anthocyanins on SN-38 mediated cytotoxicity in CT26 cells, a concentration of SN-38 inducing a potent reduction of cell viability was determined. A concentration-dependent decline in cell viability could be observed for the concentration range tested (0.1–30 nM, Supplementary Figure S3). A concentration of 10 nM SN-38 was chosen for the assessment of the combined cytotoxic properties, leading to 56.1 ± 6.0 and 39.4 ± 6.5% of viable cells in the WST-1 and SRB assay, respectively. Significant reduction in cell viability compared to the solvent control was achieved with 10 nM SN-38 in all assays testing combined exposure (Figure 2). Again, the BB extract showed strong reducing effects on cell viability, seemingly further potentiating the effect of 10 nM SN-38. The lowest percentage of viable cells was observed in combination with 200 µg/mL BB extract at 36 and 29% in the WST-1 and SRB assays, respectively (*p* < 0.001). However, according to the calculation of the independent joint action (IJA) of the BB extract and SN-38, the calculated combined effect is significantly lower than the measured cell viability in both assays. Hence, at concentrations of 100 and 200 µg/mL BB extract, an antagonistic interaction is observed (Supplementary Figure S4A,B).

The Bil extract also showed a further significant reduction in cell viability at 12.5 µg/mL in the WST-1 assay but an increase in viability of up to 116% at 100 µg/mL. Since no effect of the Bil extract on SN-38 mediated cytotoxicity is observed in the SRB assay, the inducing effect is most probably due to increased metabolic stress of the cells treated with Bil and SN-38. Except for a small synergistic tendency at 50 µg/mL Bil, observed effects are of an additive nature (Supplementary Figure S4C,D). The BC extract could significantly reduce cell protein content at 100–200 µg/mL (*p* < 0.05, Figure 2C), which was an expected additive interaction. However, the signal obtained at low concentrations (6.25–12.5 µg/mL) of BC extract indicated an antagonistic interaction in both assays (Supplementary Figure S4E,F). While the EB extract did not significantly affect SN-38-induced cytotoxicity in either assay (Figure 2A,C), the calculated combined effect tends to be lower than the measured effect for all concentrations in both assays hinting towards antagonistic interactions (Supplementary Figure S4G,H).

None of the single anthocyanins induced significant effects in the WST-1 assay (Figure 2B). However, Cy3rut significantly diminished cell protein content at 200 µM (*p* < 0.05), whereas Del3glc only showed a non-significant reducing tendency in the SRB assay (Figure 2D). While substance interactions between Cy3glc and SN-38 were of an additive nature, interactions of SN-38 with Del3glc and Cy3sam showed several antagonistic tendencies but no overlapping significances between both assays. Cy3rut was the only anthocyanin tending towards synergistic interaction potential with SN-38. However, statistical significance could only be observed in the WST-1 assay (Supplementary Figure S5).

### 2.4. Impact on DNA Damaging Properties of SN-38

**Figure 3:**
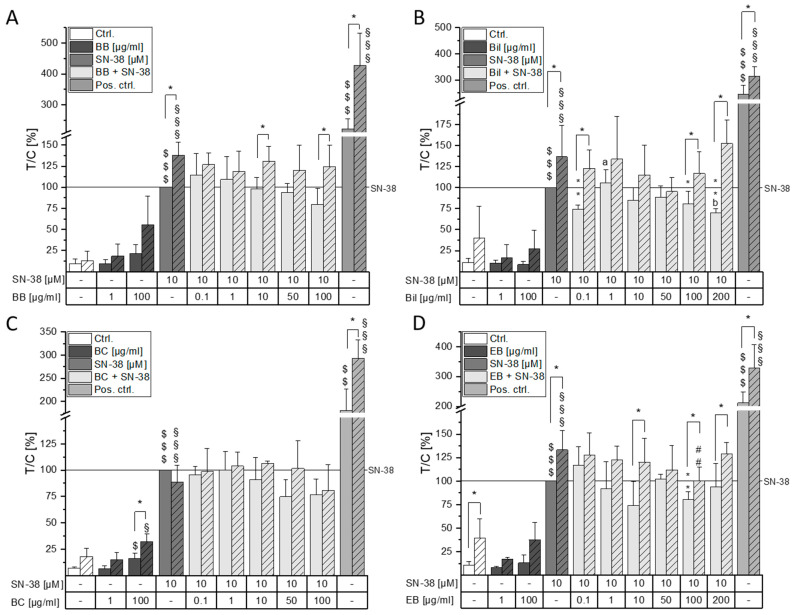
DNA-damaging properties of anthocyanin-rich (**A**) BB, (**B**) Bil, (**C**) BC, and (**D**) EB extracts alone and in combination with 10 µM SN-38. CT26 cells were pre-incubated for 30 min with the extract and then additionally challenged with SN-38 for 1 h; 1 min UV irradiation was used as a positive control. Clear and striped bars indicate an additional treatment without or with formamidopyrimidine-DNA glycosylase (FPG), respectively. Results presented are the means + SD of at least three different replicates expressed as T/C in percent relative to SN-38. Statistical significances to the respective solvent control were calculated with one- and two-sample Student’s *t*-tests and marked with $ (without FPG, $ *p* < 0.05, $$ *p* < 0.01, $$$ *p* < 0.001) and § (with FPG, § *p* < 0.05, §§§ *p* < 0.001). Statistical differences to SN-38 were calculated with one-sample Student’s *t*-test (without FPG, * *p* < 0.05, ** *p* < 0.01) or two-sample Student’s *t*-test (with FPG, ^##^ *p* < 0.01). One-way ANOVA with post hoc Bonferroni test was used to calculate statistical differences of the applied concentrations (*p* < 0.05, a, b; e.g., the letter a represents significance compared to the lowest concentration of 0.1 µg/mL).

**Figure 4:**
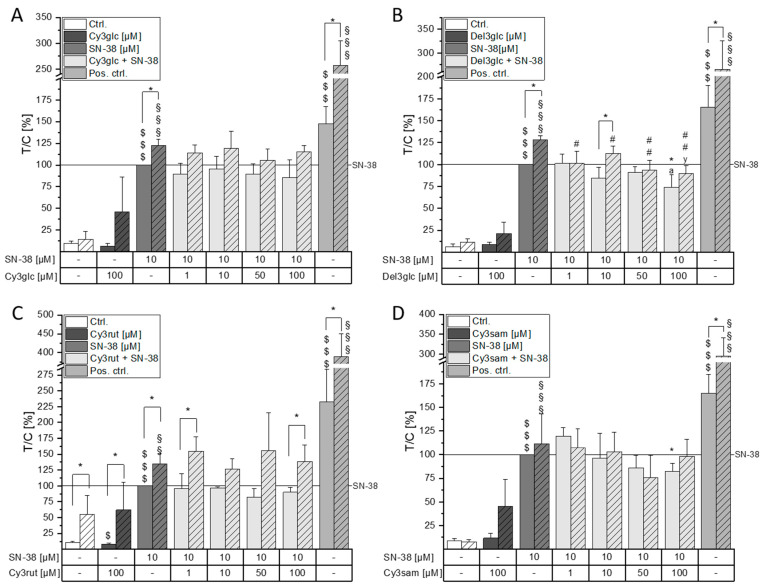
Influence of (**A**) Cy3glc, (**B**) Del3glc, (**C**) Cy3rut, and (**D**) Cy3sam on SN-38-induced genotoxicity. For the comet assay, cells were pre-incubated with the respective anthocyanin for 30 min and subsequently additionally challenged with 10 µM SN-38 for 1 h. Irradiation with UV light for 1 min served as positive control. Striped bars represent samples additionally treated with FPG. Data presented are the means + SD of at least three independent replicates shown as T/C in percent relative to SN-38. Statistical differences to the respective solvent control were calculated with one- or two-sample Student’s *t*-tests and indicated with $ (without FPG, $ *p* < 0.05, $$$ *p* < 0.001) or § (with FPG, §§ *p* < 0.01, §§§ *p* < 0.001). Statistical differences compared to SN-38 treated without (* *p* < 0.05) or with FPG (# *p* < 0.05, ## *p* < 0.01) were calculated with one- or two-sample Student’s *t*-tests, respectively. Significances among the tested concentrations were calculated with one-way ANOVA via a post hoc Bonferroni test (*p* < 0.05) and indicated with a–c (without FPG; e.g., the letter a represents significance compared to the lowest concentration of 1 µM) or x–z (with FPG).

As a topo I poison, SN-38 stabilizes the DNA/topo I covalent complex, which leads to the persistence of DNA single-strand breaks, but double-strand breaks could also occur due to collision with the replication fork [21]. It was therefore of interest if the anthocyanin-rich berry preparations can modify SN-38-induced genotoxic effects. For this purpose, a concentration range from 0.1–10 µM SN-38 was tested for its DNA-damaging properties. A concentration-dependent increase in tail intensity could be observed (Supplementary Figure S6). Although a statistically significant induction of tail intensity was already observed with a concentration of 0.5 µM SN-38, the highest concentration tested (10 µM) induced the signal to 10.6 ± 1% and was hence chosen for further testing to facilitate observation of combinatory effects in both directions. Cells were thus pre-incubated with the anthocyanin-rich extracts or the respective single anthocyanins and then co-incubated with 10 µM SN-38 for one hour. To ameliorate comparability of the results, combinatory comet assays were evaluated as test over control (T/C) of tail intensity relative to 10 µM SN-38 without additional formamidopyrimidine-DNA glycosylase (FPG) treatment. SN-38, as well as the positive control (1 min UV-B irradiation), significantly increased DNA strand breaks when compared to the respective solvent control sample ($$$ *p* < 0.001 without FPG, §§§ *p* < 0.001 with FPG, Figure 3 and Figure 4). Moreover, FPG treatment significantly further induced DNA damage in the positive control. Pre- and co-incubation with the BB and BC extract did not significantly affect SN-38-induced DNA damage. However, a tendency to reduce DNA strand breaks can be observed at the highest concentration tested (100 µg/mL, Figure 3A,C). For the BB and BC extracts, 200 µg/mL could not be tested in the comet assay because cell viability was below 80% as evaluated through a trypan blue exclusion test. Furthermore, 100 µg/mL BC extract alone significantly increased DNA damage when compared to the solvent control ($, § *p* < 0.05). The Bil extract, on the contrary, did not affect DNA strand breaks when incubated alone but significantly reduced SN-38-induced DNA damage at the lowest and the two highest concentrations (0.1, 100–200 µg/mL, Figure 3B). However, these effects were only observed in samples without additional FPG treatment. Only the EB extract significantly reduced both FPG-treated (## *p* < 0.01) and non-FPG-treated (** *p* < 0.01) DNA damage at 100 µg/mL (Figure 3D).

To determine whether the effects observed with the anthocyanin-rich extracts can be attributed to their respective most abundant single anthocyanin, CT26 cells were pre-incubated with Cy3glc, Del3glc, Cy3rut, and Cy3sam and subsequently additionally challenged with 10 µM SN-38. The main constituents of the BB and the BC extracts—Cy3glc and Cy3rut, respectively—did not show any significant effects compared to the SN-38 control in the concentration range tested (1–100 µM, Figure 4A,C). When the anthocyanins were incubated without SN-38, no significant induction of DNA damage could be observed at 100 µM. Del3glc, in contrast, significantly reduced SN-38-induced DNA damage at 100 µM down to 74 ± 15% (* *p* < 0.05, Figure 4B). Interestingly, Del3glc also reduced DNA strand breaks in FPG-treated samples in all concentrations tested (#, ## *p* < 0.05, <0.01). In case of Cy3sam, 100 µM significantly reduced SN-38 mediated DNA damage in samples not treated with FPG and additionally showed a tendency towards reduction already at 50 µM (Figure 4D).

### 2.5. Impact on Cell Adhesion

**Figure 5:**
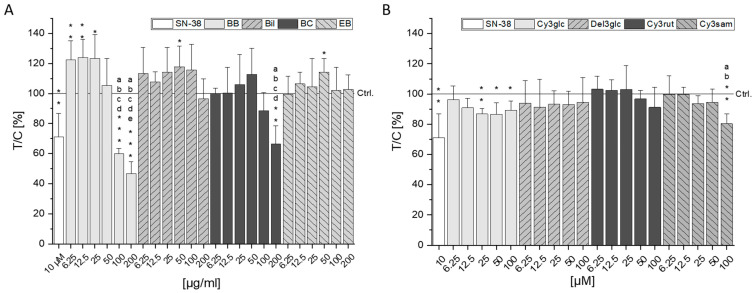
Effects of anthocyanin-rich extracts (**A**) and single anthocyanins (**B**) on cell adhesion in CT26 cells measured with the crystal violet assay. Cells were incubated with the test substances for 2 h, and adhered cells were fixed and stained according to the assay protocol. Results presented are the means + SD of at least three independent biological replicates measured in technical triplicates relative to the solvent control (Ctrl., 0.6% DMSO). Statistical differences were calculated with one-sample Student’s *t*-tests (* *p* < 0.05, ** *p* < 0.01, *** *p* < 0.001) and one-way ANOVA via a posthoc Bonferroni test (*p* < 0.05, a–e; e.g., the letter a represents significance compared to the lowest concentration of 6.25 µg/mL or µM).

The impact of increasing concentrations of berry extracts and single anthocyanins on adhesion of CT26 cells to standard tissue culture material was investigated after 2 h of exposure via crystal violet staining (Figure 5). While low concentrations of BB extract showed increased cell adhesion compared to the solvent control, 100 and 200 µg/mL BB significantly decreased cell adhesion to 60 ± 3 and 47 ± 8%, respectively (*p* < 0.001, Figure 5A). At 50 µg/mL of both Bil and EB extract, a tendency towards increased cell adhesion could be observed (*p* < 0.05). However, other concentrations of these extracts could not affect the adhesion of CT26 cells. Furthermore, 200 µg/mL BC extract reduced cell adhesion to the same level as 10 µM SN-38 (*p* < 0.01). However, effects observed at 200 µg/mL BB and BC extracts might be the results of mediated cytotoxicity, as trypan blue exclusion after 1.5 h in the comet assay already showed a viability below 80% for both extracts but not for 10 µM of SN-38.

Single anthocyanins could only marginally affect cell adhesion, if at all. Cy3glc, starting from a concentration of 25 µM, significantly reduced cell adhesion to about 87–89%. Furthermore, 100 µM Cy3sam showed significantly fewer adhered cells compared to the control (*p* < 0.01).

## 3. Discussion

In the present study, anthocyanin-rich extracts of blackberry, bilberry, black currant, and elderberry (BB, Bil, BC, and EB, respectively) were analyzed for their anthocyanin composition and then tested for their inhibitory potential in murine CRC cells. Corroborated by a body of existing literature, the four berry extracts differed significantly in their anthocyanin profile. A great variety of anthocyanins is reported for bilberry extracts, where between 15 and 18 different ones could be identified depending on the detection method and available standards [22,23,24]. Comparable to the Bil extract, several groups reported Del3glc and Cy3glc as the most prevalent anthocyanins in bilberry [23,24,25]. However, Del3-galactoside—which ranks third most abundant anthocyanin in our extract—was also reported as a main component [22,26]. In agreement with the literature, the BB extract only consists of cyanidin-based anthocyanins with Cy3glc (88.2% of total anthocyanins) as the major compound and small amounts of Cy3dioxglc, Cy3malglc, and Cy3xyl [23,27,28]. Cy3rut, representing 3% of total anthocyanins in the BB extract, was not detected in aforementioned publications, but Fan-Chiang et al. reported a mean percentage of 10.2 Cy3rut in 51 blackberry samples from different American and international origins [29]. The seeming discrepancy most probably arises from either the lack of available standards or from a variation in pH, temperature, or sunlight during the growth of the berries since it is generally known that these factors influence anthocyanin synthesis [30]. The BC extract comprises roughly 40% of each Cy3rut and Del3rut and only small amounts of petunidin- and peonidin-3-*O*-rutinoside. Consistent with the available literature, Del3glc (11%) and Cy3glc (5%) were also identified [22,26,31,32]. The main anthocyanin in elderberry is reported to mostly be Cy3sam [31,33,34]. However, Cy3sam5glc—ranking second place in the EB extract—has also been reported as a main component [35].

While Bil, BC, and EB extracts were only slightly cytotoxic in CT26 cells, the BB extract showed potent cytotoxic properties leading to a concentration-dependent reduction in cancer cell growth. However, these effects do not appear to be attributable to its main anthocyanin, Cy3glc, since no cytotoxicity was evident up to 50 µM (200 µg/mL BB extract contain ~29 µM of Cy3glc). Concomitantly, Evora et al. reported cytotoxic effects of 100 µM blackberry anthocyanins but not of up to 100 µM Cy3glc in HaCat keratinocytes after 48 h [28]. Since neither of the observed effects from BB extract could be attributed to the main anthocyanin Cy3glc, it is hypothesized that synergistic interactions of other extract components contribute to the potent cytotoxic effects. The induction of DNA strand breaks could be ruled out as a potential underlying mechanism for BB cytotoxicity since no difference to the solvent control could be observed (Figure 3A). A non-significant tendency to induce FPG-sensitive sites was observed with 100 µM BB hinting towards potential pro-oxidative properties of the extract. In combination with the topo I poison SN-38, BB extract further decreased cell viability. However, this interaction was found to be of an antagonistic nature according to calculation of IJA. Hence, while BB does not per se protect the cancer cells from SN-38-induced damage, the measured combined effect is lower than the theoretically calculated combined effect. When investigating a commercially available bilberry extract, Aichinger et al. showed antagonistic interactions with the chemotherapeutic drug erlotinib over the whole tested concentration range [36]. While little to no cytotoxic effect could be observed with the Bil extract, the nature of interaction between Bil and SN-38 was overall found to be additive. This points out the complexity of food–drug interactions that are dependent not only on the drug’s mechanism of action but also on the polyphenol and anthocyanin profile of the extracts used. Furthermore, less tail intensity could be observed when co-incubating Bil and SN-38 in the comet assay at the lowest and highest concentrations. The Bil extract, therefore, seemingly has the potential to reduce SN-38 efficacy in CT26 cells. However, this effect did not seem to be attributable to the main anthocyanin Del3glc alone but might rather be the result of a mixture effect. It could be hypothesized that Del3glc in combination with other topo-I-inhibiting substances contained in the Bil extract interferes with SN-38 mediated topo I poisoning, leading to a reduction in strand breaks. Interestingly, FPG-sensitive sites are not affected by this reducing effect of Bil, which could be an indicator of oxidative stress mediated by the Bil extract. FPG-sensitive sites were only reduced significantly by 1–100 µM Del3glc and 100 µg/mL EB extract but were not affected by any of the other tested anthocyanins or extracts, leading to the hypothesis that Del3glc and EB might be able to reduce oxidative stress. Indeed, anthocyanin constituents of EB, Cy3sam, and Cy3sam5glc, have been identified as anti-oxidative in Caco-2 cells [33]. Interestingly, anthocyanins alone did not affect tail intensity of CT26 cells even though the respective anthocyanidins induced DNA damage starting from 50 µM in HT29 cells [17,37]. Overall, induction of DNA damage could not be identified as an underlying mechanism to the observed cytotoxicity of the extracts. The induction of apoptosis or cell cycle arrest has been proposed for bilberry- and grape-pomace-retrieved anthocyanin extracts [8,38] and should be further investigated with the presented extracts.

Cell adhesion plays a fundamental role in multiple processes of cell regulation. Furthermore, adhesion changes are viewed as defining incidents in cancer cell development and progression since during the process of metastasis, cancer cells travelling through the blood stream need to first detach from their site of origin and finally invade and adhere to new surroundings at the metastatic niche [18,39]. It was therefore of great interest if anthocyanin-rich berry extracts and their respective major anthocyanins could interfere with the cell adhesion behavior of CT26 cells. As a point of reference, 10 µM SN-38 was used and diminished cell adhesion significantly to 71 ± 16% after 2 h of incubation. Similar experiments with 40 µg/mL (~68 µM) of the parent compound CPT-11 led to about 75% of adhered cells compared to the control in human colon cancer HCT116 cells, whereas this concentration increased cell adhesion of normal human colonic fibroblasts 1.4-fold [40]. While low concentrations of the BB extract also slightly increased CT26 adhesion, the highest concentrations of 100–200 µg/mL potently diminished the number of adhered cells, even to a greater extent than 10 µM SN-38. A dose-dependent reduction in cell-matrix adhesion to type I collagen for Cy3glc (25–100 µM) after 24 h pre-incubation was reported in A549 cells [41]. CD40-induced ICAM-1 and VCAM-1 secretion was dose-dependently inhibited by Cy3glc (1–100 µM) in HUVEC cells [42]. Furthermore, 20 µg/mL (~44 µM) Cy3glc as well as a blackberry anthocyanin fraction reduced LPS and conditioned medium-induced ICAM-1 expression in RAW264.7 and Caco-2 cells, respectively [19]. While no dose-dependency was observable in the present study, Cy3glc already diminished cell adherence in concentrations present in high concentrations of the BB extract (200 µg/mL contain 29.9 µM Cy3glc). Therefore, one might speculate that Cy3glc interacting with other compounds comprising the BB extract is a driving factor for the potent inhibition of cell adhesion observed in CT26 cells. However, at the highest concentration of 200 µg/mL, an overlay with cytotoxic properties of the BB extract can be expected because this concentration showed a cell viability below 80% after 1.5 h incubation in the trypan blue exclusion assay performed within the comet assay. Similar to the Bil extract, a blueberry extract did not interfere with adhesion properties of either CT26 or HeLa cells up to 50 mg/mL gallic acid equivalents [43]. Moreover, the main anthocyanin of the Bil extract, Del3glc, did not affect cell adhesion. Anthocyanins obtained from black soybean, containing 20% Del3glc, could dose-dependently diminish TNF-induced VCAM-1 induction. However, this effect might be attributed to the 72% of Cy3glc in this mixture [20]. Despite reducing ICAM-1 expression at a concentration of 20 µg/mL [19], Cy3rut did not interfere with cell adhesion either in the present study or in A549 cells after 24 h pre-incubation [41]. Hence, the diminished cell adhesion mediated by the BC extract is not attributable to its main anthocyanin but rather is the result of an overlay with cytotoxicity at 200 µg/mL since a cell viability below 80% could be observed within trypan blue exclusion after 1.5 h. To the best of our knowledge, effects of Cy3sam and EB extract on cell adhesion of mammalian cells have not yet been reported. However, Antolak et al. showed reduced cell adhesion of bacterial cells (*Asaia* spp. strains) to different surfaces by co-incubation with elderberry juice [44]. Comparatively, the EB extract used in the present study did not diminish adhesion properties of CT26 cells, while its main anthocyanin, Cy3sam, significantly reduced the number of adhered cells to 80 ± 7% at 200 µM. However, this concentration exceeds the amount of Cy3sam present in 200 µg/mL EB extract fivefold.

Overall, out of the four extracts tested, the BB extract inhibited cancer cell growth and adherence most potently in a concentration-dependent manner. Furthermore, BB extract enhanced the cytotoxic efficacy of SN-38 even though interactions were found to be antagonistic in nature. However, an induction of DNA strand breaks could not be identified as underlying mechanism, since neither the BB extract alone nor in combination with SN-38 increased DNA damage. Within this study, the inhibitory potential of the extracts on CT26 colon cancer cells can be ranked BB >> BC ≥ Bil ≈ EB. In general, the effects observed when studying the extracts could not be attributed to their single main anthocyanin and, presumably, result from synergistic interactions of extract constituents rather than single bioactive substances. Due to the low reported systemic bioavailability of anthocyanins, local interactions with the GIT are of special interest. Future experiments should aim to elucidate the potential specificity of the observed effects to cancer cells and investigate their safe use on non-tumor cells. Furthermore, the relevance of the observed effects in vivo should be investigated.

## 4. Materials and Methods

### 4.1. Materials

The single anthocyanins Cy3glc (purity > 96%), Del3glc (>95%), Cy3rut (>96%), and Cy3sam (>95%) were obtained from Extrasynthèse (Genay, France). Catalase was purchased from Merck (Vienna, Austria), and SN-38 (>98%) was purchased from Tocris (Bristol, UK). Anthocyanin-rich extracts were obtained as described below.

### 4.2. Juice Manufacturing

All extracts were prepared from 100% berry juices. Organic bilberry juice (*Vaccinium myrtillus*) was provided from Bayernwald Früchteverwertung KG (Hengersberg, Germany) in 5 L bag-in-boxes. Frozen blackberries, black currants, and elderberries were purchased loose rolling from Trautner Fruit Trade (Graefenberg, Germany). Frozen fruits were thawed and heated to an enzymation temperature of 50 °C in a ploughshare mixer (Loedige, Paderborn, Germany). After mash depectinization (200 mL/t Fructozym Color, Erbsloeh, Geisenheim, Germany), blackberry and elderberry juices were extracted on a rack and frame press (Wahler, Stuttgart, Germany). The resulting juices were again depectinized, filtered without any previous fining steps, and subjected freshly to adsorber resin treatment. Black currants were extracted under vacuum (Vaculiq, GEA-Group, Duesseldorf, Germany); the finished juice was pasteurized and stored in sterilized 30 L stainless steel kegs.

### 4.3. Extract Manufacturing

Juice polyphenols were retained on glass columns packed with adsorber resins. Bilberry juice (20 L) was loaded on a Pilot column (750 × 200 mm, YMC, Dinslaken, Germany) packed with 15 L of SP70 resin (Resindion, Binasco, Italy) and rinsed with 40 L of distilled water to remove undesired water-soluble compounds such as sugars, fruit acids, and minerals. The polyphenolic fraction containing anthocyanins was then desorbed with 20 L of ethanol. Blackberry, black currant, and elderberry juices were subjected to a smaller BPG-100 glass column (1000 × 100 mm, GE Healthcare Life Sciences, Freiburg, Germany) packed with 5 L of ADS5000 resin (Chemra, Trier, Germany) and processed analogously. Liquid ethanolic adsorber resin extracts were concentrated in a Hei-VAP industrial rotation evaporator (Heidolph, Schwabach, Germany), transferred into the aqueous phase, and freeze dried (Beta 2-8 LD+, Christ, Osterode, Germany). The hygroscopic powders were stored under dry, cool, and dark conditions.

### 4.4. HPLC Analysis of Anthocyanins

Freeze-dried adsorber resin extracts from berry juices were dissolved in HPLC start gradient (5% formic acid/methanol 90/10 *v*/*v*) in concentrations of 0.5 and 1.0 g/L. Chromatographic separation was achieved on an Accela HPLC system (Thermo Fisher, Dreieich, Germany) using a 150 × 2 mm i.d., 3 µm LUNA C18 column (Phenomenex, Aschaffenburg, Germany) protected with a guard column of the same material. Injection volume was 4 µL. Elution conditions were: 250 µL/min flow rate at 40 °C; Solvent A was 5% formic acid (ULC/MS grade, Promochem, Wesel, Germany); Solvent B, methanol (gradient grade, Roth, Karlsruhe, Germany); 1 min isocratic conditions with 10% B, linear gradient from 10 to 40% B in 12 min, followed by washing for 7 min with 100% B and re-equilibrating the column. Quantitation was carried out using peak areas (520 nm trace) from external calibration via the reference substance Cy3glc. Anthocyanin analysis was carried out in duplicate.

### 4.5. Electrospray Ionization (ESI)-MS of Anthocyanins

For mass detection the Accela HPLC system was coupled to a Thermo Finnigan LXQ mass spectrometer (Thermo Fisher, Dreieich, Germany) equipped with an ESI source and an ion trap mass analyzer. The whole system was controlled by Xcalibur software (version 2.2). For anthocyanins, the mass spectrometer was operated in the positive mode with the following conditions: source voltage 4.5 kV; capillary voltage 32 V; capillary temperature 275 °C; collision energy 30 (MS^2^); and 33% (MS^3^). All anthocyanins could be assigned safely according to their mass spectra.

### 4.6. Cell Culture

Murine CT26 colon carcinoma cells (ATCC, Manassas, VA, USA) were cultured in Dulbecco’s Modified Eagle’s Medium: Nutrient Mixture F-12 Ham 1:1 medium (Merck, Vienna, Austria) supplemented with 10% fetal calf serum and 1% penicillin/streptomycin (Thermo Fisher, Vienna, Austria). Cells were kept in a humidified atmosphere at 37 °C with 5% CO_2_ and passaged twice per week.

### 4.7. Treatment and Dosage Information

Effects of berry extracts and single anthocyanins alone were investigated in concentration ranges between 6.25–200 µg/mL or 6.25–100 µM, respectively. While the concentration ranges for the extracts are considered easily reachable through a healthy diet, higher concentrations of single anthocyanins could probably be acquired via the ingestion of fortified preparations, such as food supplements. Furthermore, cells were pre-incubated for 30 min with anthocyanins or extracts to allow uptake by cells before co-incubation with the more lipophilic SN-38 to assess potential combinatory effects. All substances were dissolved in DMSO and stored in small aliquots at −80 °C (anthocyanins, extracts) or −20 °C (SN-38) to avoid repeated freeze–thaw cycles. Final solvent concentration during incubation did not exceed 0.6% DMSO. All incubations were performed in the presence of 100 U/mL catalase, which was dissolved freshly in phosphate-buffered saline (PBS), to suppress the accumulation of H_2_O_2_.

### 4.8. Coupled WST-1 and SRB Assay

Cells (1500 cells/well) were seeded in 96-well plates and grown for 24 h. They were either incubated with berry extracts or anthocyanins alone for 72 h or after short-time pre-incubation with the extracts/anthocyanins (30 min), and cells were additionally challenged with SN-38 for 72 h. Furthermore, cells treated with normal cell culture medium were compared to the solvent control to exclude potential reduction of cell viability by treatment with 0.6% DMSO. Afterwards, a WST-1 solution (abcam, Cambridge, UK) was added to the cells and metabolically active cells cleaved the tetrazolium salt WST-1 to formazan via mitochondrial dehydrogenases. The absorption of the produced formazan dye was measured after 45 min with a microplate reader (450 nm; reference wavelength: 650 nm) and gave an insight on cytotoxicity. Thereafter, cells were washed with PBS and fixed with trichloroacetic acid (5%, *w*/*v*) solution at 4 °C for 1 h. After washing the cells and letting them dry overnight, protein content was stained with SRB solution for 1 h (Alfa Aefsar, Haverhill, MA, USA). Excess dye was rinsed away with water followed by 1% acetic acid solution, and the plate dried overnight. The dye was then dissolved under basic conditions with 10 mM TRIS Base buffer (Carl Roth, Karlsruhe, Germany), and absorbance was measured at 570 nm with a microplate reader [45].

### 4.9. Interaction Analysis

Mathematical models are often used to ameliorate interpretation of substance interactions. In this study, the widely used model of independent joint action was applied to calculate the expected additive value of compound combinations with Formula (1):

(1) $$f_{ab}=f_{a}+f_{b}-f_{a}\times f_{b}$$

*f_ab_* stands for the expected combined effect/and *f_a_*, *f_b_* are the measured effects of the single substances. The calculated combined effect (=1 − *f*_ab_) is then compared to the measured combined effect with a two-sample Student’s *t*-test to determine if the interaction is antagonistic, additive, or synergistic in nature [46,47].

### 4.10. Comet Assay

Single-cell electrophoresis (comet assay) was performed according to the guidelines of Tice et al. [48]. Briefly, 2.2 × 10^5^ cells were seeded into 35 mm petri dishes and grown for 24 h. Cells were pre-incubated with the anthocyanins or extracts for 30 min, followed by co-incubation with SN-38 for 60 min; 1 min of UV-B irradiation served as a positive control. Afterwards, cells were washed twice with PBS and trypsinized, and cell viability was determined via trypan blue exclusion. Only samples with a viability of over 80% were processed, and two slides were prepared for each sample. A cell count of 30,000 cells was embedded in low-melting agarose (0.8%, Bio-Rad, Vienna, Austria) in phosphate buffer onto a normal-melting agarose gel (0.8%, Bio-Rad, Vienna, Austria) in duplicate. Cells were lysed overnight at 4 °C in a lauroyl-sarcosinate- (Merck, Vienna, Austria), Triton-X 100 (Carl Roth, Karlsruhe, Germany) and DMSO- (Carl Roth, Karlsruhe, Germany) containing buffer. Half of the slides were then treated with FPG (New England Biolabs, Frankfurt, Germany) at 37 °C for 30 min. After 20 min of equilibration in electrophoresis buffer (pH 13, NaOH, EDTA), the electrophoretic run was conducted at 0 °C, 300 ± 3 mA and 25 V (0.028 V/cm^2^) for 20 min. Slides were washed with neutralization buffer (TRIS, pH 7.5, Carl Roth, Karlsruhe, Germany) and stained with DNA-intercalating ethidium bromide solution (0.02 mg/mL, Carl Roth, Karlsruhe, Germany). A Zeiss Axioskop (λ_ex_ 546 ± 1 nm, λ_em_ ≥ 590 nm) was utilized for microscopic analysis. The “Comet Assay IV” software (version 4.2.1, Perceptive Instruments, Suffolk, UK) was used for image analysis, and 50 nuclei per gel pad were scored, resulting in 100 scored nuclei per condition. Tail intensity values were used for test over control (T/C) evaluation in percent; 10 µM SN-38 without FPG treatment was set to 100%.

### 4.11. Cell Adhesion Assay

Cells at a density of 4 × 10^4^ cells/well were seeded into 96-well plates containing increasing concentrations of extracts or single anthocyanins and incubated for 2 h at 37 °C. Wells were washed with pre-warmed PBS to remove non-adherent cells and incubated for 15 min with 0.1% crystal violet (Merck, Vienna, Austria) solution in 10% ethanol at 4 °C. The staining solution was discarded, and the wells were rinsed with tap water to remove excess dye. After the plate had dried, the crystals were dissolved in ethanol containing 1% acetic acid, and absorbance was measured at 595 nm with a microplate reader.

### 4.12. Statistical Analysis

Outliers were identified and eliminated via a Nalimov outlier test. Data presented are the mean + SD of at least three independent biological replicates measured in technical duplicates to triplicates. Normal distribution was determined with the Shapiro–Wilk normality test. Statistical significances were determined with one-way ANOVA via a post hoc Bonferroni test as well as one- and two-sample Student’s *t*-tests using the software OriginPro 2021. Values were considered statistically significant if *p* < 0.05, <0.01, <0.001.

## Figures and Tables

**Table 1:** Anthocyanin profile of the four berry extracts in g/kg extract and percentage of total anthocyanins. The main anthocyanins in each extract are marked grey.

Anthocyanins	Bilberry		Black Currant		Blackberry		Elderberry	
	(g/kg)	%	(g/kg)	%	(g/kg)	%	(g/kg)	%
cyanidin-3-*O*-glucoside	13.8	13.5	2.4	5.3	67.3	88.5	-	-
cyanidin-3-*O*-arabinoside	7.5	7.3	-	-	-	-	-	-
cyanidin-3-*O*-galactoside	9.1	8.9	-	-	-	-	-	-
cyanidin-3-*O*-rutinoside	-	-	18.2	39.4	2.3	3.0	-	-
cyanidin-3-*O*-sambubioside	-	-	-	-	-	-	56.6	72.9
cyanidin-3-*O*-sambubioside-5-*O*-glucoside	-	-	-	-	-	-	21.0	27.1
cyanidin-3-*O*-xyloside	-	-	-	-	1.5	1.9	-	-
cyanidin-3-*O*-(6″-*O*-malonyl)-glucoside	-	-	-	-	1.5	2.0	-	-
cyanidin-3-*O*-(6″-*O*-dioxalyl)-glucoside	-	-	-	-	3.5	4.6	-	-
delphinidin-3-*O*-glucoside	17.1	16.7	5.1	11.0	-	-	-	-
delphinidin-3-*O*-arabinoside	10.5	10.2	-	-	-	-	-	-
delphinidin-3-*O*-galactoside	12.6	12.3	-	-	-	-	-	-
delphinidin-3-*O*-rutinoside	-	-	19.1	41.3	-	-	-	-
malvidin-3-*O*-glucoside	7.8	7.6	-	-	-	-	-	-
malvidin-3-*O*-arabinoside	1.2	1.1	-	-	-	-	-	-
malvidin-3-*O*-galactoside	2.6	2.5	-	-	-	-	-	-
peonidin-3-*O*-glucoside	4.2	4.1	-	-	-	-	-	-
peonidin-3-*O*-galactoside	0.6	0.6	-	-	-	-	-	-
peonidin-3-*O*-rutinoside	-	-	0.5	1.1	-	-	-	-
petunidin-3-*O*-glucoside	9.5	9.3	-	-	-	-	-	-
petunidin-3-*O*-arabinoside	2.0	2.0	-	-	-	-	-	-
petunidin-3-*O*-galactoside	3.8	3.7	-	-	-	-	-	-
petunidin-3-*O*-rutinoside	-	-	0.9	1.9	-	-	-	-
Total	102.5	100	46.3	100	76.0	100	77.6	100

## Data Availability

The majority of data are presented in the publication and the Supplementary Materials. If further data are desired, they can be requested from the corresponding author.

## Supplementary Materials

The following supporting information can be downloaded at: https://www.mdpi.com/article/10.3390/molecules28237684/s1, Figure S1: Chemical structures of anthocyanidins (A) cyanidin (Cy), (B) delphinidin (Del), and the sugars bound to position 3 of the anthocyanins (C) glucoside (glc), (D) rutinoside (rut), and (E) sambubioside (sam); Figure S2: Chromatograms of (A) elderberry (EB), (B) bilberry (Bil), (C) black currant (BC), and (D) blackberry (BB); Table S1: Peak annotation of the chromatograms shown in Figure S2; Figure S3: Cytotoxic properties of different concentrations of SN-38 assessed by the coupled WST-1 and SRB assay; Figure S4: Independent joint action calculation from cytotoxic effects of blackberry, bilberry, black currant, and elderberry extracts and SN-38 assessed with the WST-1 and SRB assays; Figure S5: Independent joint action calculation from cytotoxic effects of Cy3glc, Del3glc, Cy3rut, and Cy3sam and SN-38 assessed with the WST-1 and SRB assays; Figure S6: DNA-damaging properties of different concentrations of SN-38 assessed with the comet assay.
