# Supplementary material for: In Vitro Inhibitory Potential of Different Anthocyanin-Rich Berry Extracts in Murine CT26 Colon Cancer Cells
Source: Molecules. 2023 Nov 21;28(23):7684. doi: 10.3390/molecules28237684 (PMC10707341; doi:10.3390/molecules28237684)
Supplement: Supplementary file 1 [file molecules-28-07684-s001.zip › molecules-2720833-supplementary.pdf]

Supplementary information for

In vitro inhibitory potential of different anthocyanin-rich berry extracts in murine CT26 colon cancer cells

Cornelia Schmutz [1,2], Frank Will [3], Elisabeth Varga [1,#], Carola Jaunecker [4], Gudrun Pahlke [1], Walter Berger [4] and Doris Marko [1,*]

[1] Department of Food Chemistry and Toxicology, Faculty of Chemistry, University of Vienna; Währingerstraße 38-40, 1090 Vienna, Austria; cornelia.schmutz@univie.ac.at (CS), gudrun.pahlke@univie.ac.at (GP)

[2] Doctoral School in Chemistry; University of Vienna; Währingerstraße 42, 1090 Vienna, Austria

[3] Department of Beverage Research, Hochschule Geisenheim University; P.O. Box 1154, 65366 Geisenheim, Germany; Frank.Will@hs-gm.de (FW)

[4] Center for Cancer Research and Comprehensive Cancer Center, Medical University of Vienna; Borschkegasse 8a, 1090 Vienna, Austria; carola.jaunecker@meduniwien.ac.at (CJ), walter.berger@meduniwien.ac.at (WB)

[#] Current address: Unit of Hygiene and Technology of Food; Institute of Food Safety, Food Technology and Veterinary Public Health; University of Veterinary Medicine Vienna; Veterinärplatz 1, 1210 Vienna, Austria; elisabeth.varga@vetmeduni.ac.at (EV)

* Correspondence: doris.marko@univie.ac.at (DM)

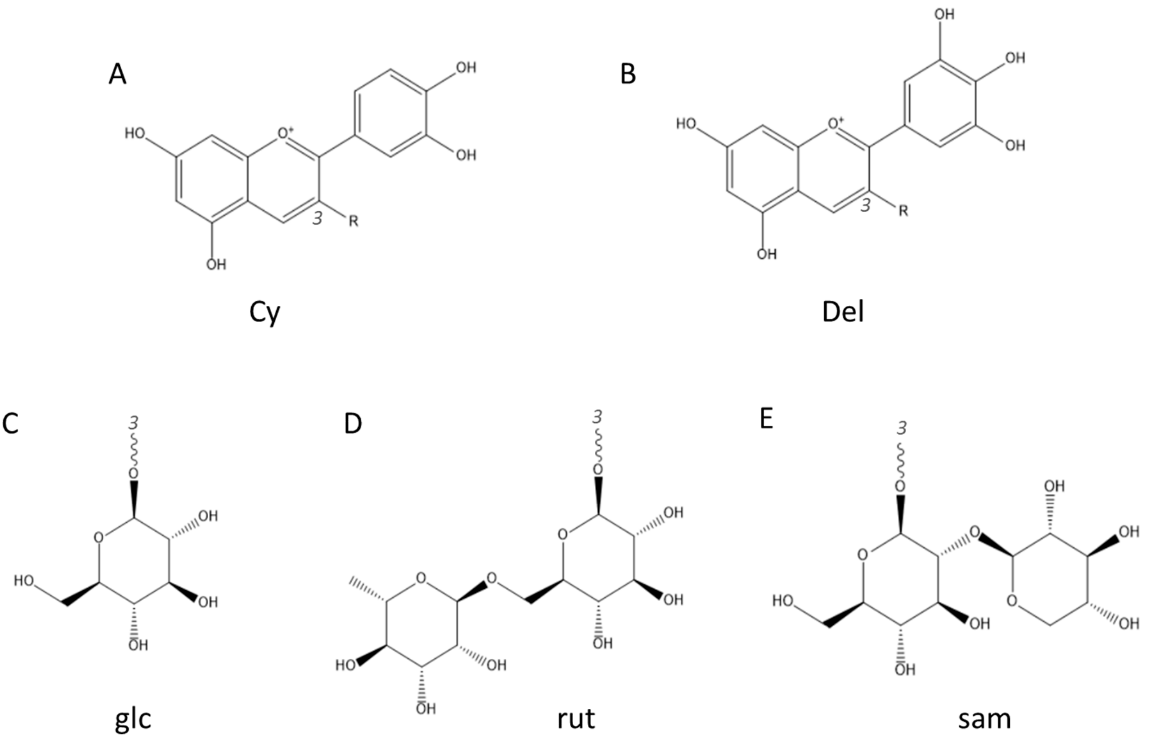

Figure S1: Chemical structures of anthocyanidins (**A**) cyanidin (R=OH, Cy), (**B**) delphinidin (R=OH, Del) and the sugars bound to position 3 of the anthocyanidins (**C**) glucoside (glc), (**D**) rutinoside (rut) and (**E**) sambubioside (sam).

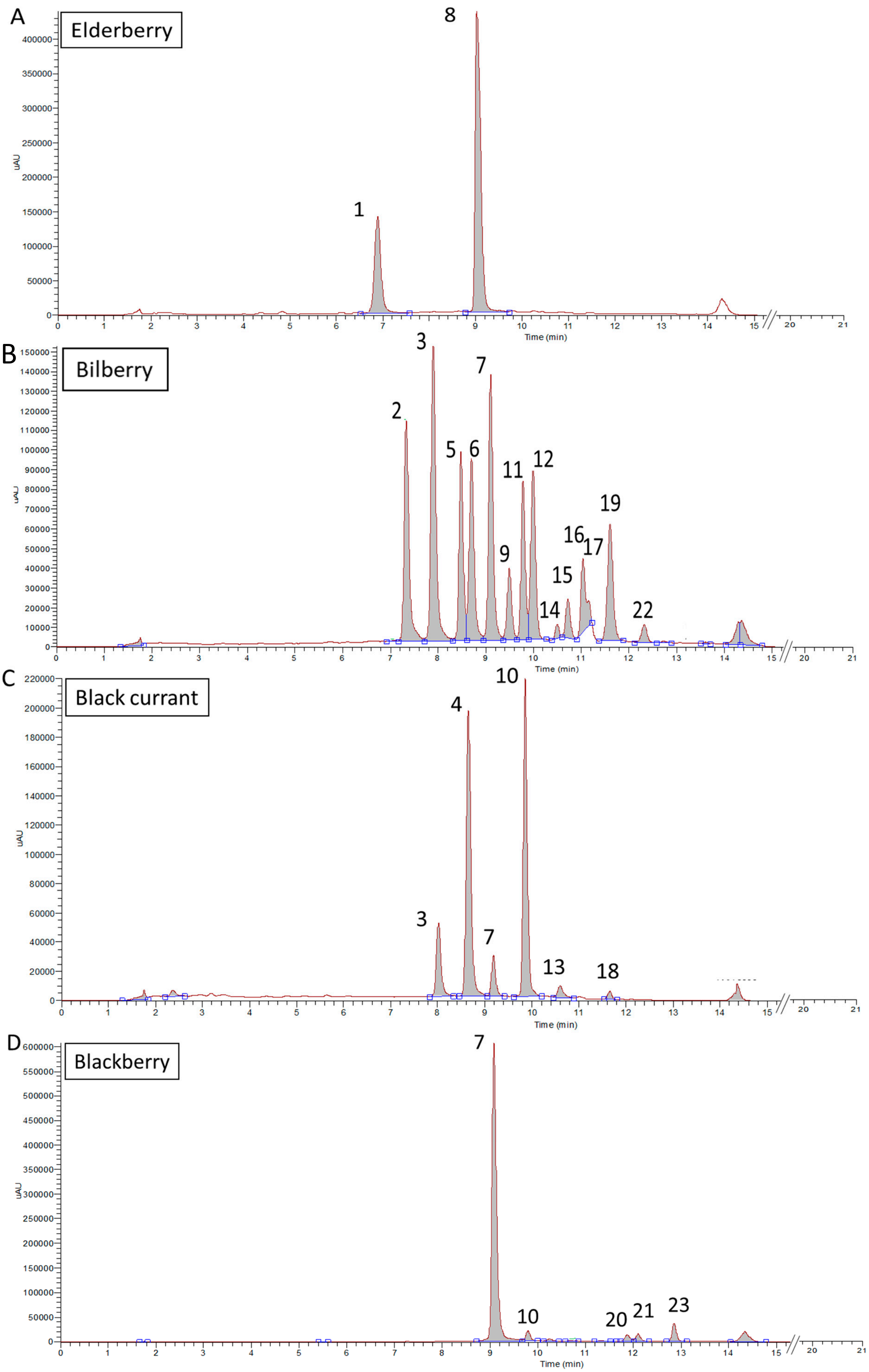

A
Elderberry
B
Bilberry
C
Black currant
D
Blackberry
uAU
Time (min)
1
8
2
3
5
6
7
9
11
12
14
15
16
17
19
22
3
4
7
10
13
18
7
10
20
21
23

Figure S2: Chromatograms of (**A**) elderberry (EB), (**B**) bilberry (Bil), (**C**) black currant (BC) and (**D**) blackberry (BB). Peak annotation is described in Table S1 below. Chromatographic separation was performed using a 3 µm LUNA C18 column (150x2 mm) with an injection volume of 4 µl. Solvent A and B were 5 % formic acid and methanol, respectively. After 1 min isocratic condition with 10 % B, a linear gradient from 10-40 % B for 12 min followed. Ultimately, washing with 100 % B was performed for 7 min. Peaks were assigned according to their mass spectra and retention times.

Table S1: Peak annotation of the chromatograms shown in Figure S2. Retention times (RT) and mass to charge (*m*/*z*) values of parent compounds and specific fragments used for analysis and assignation of single anthocyanins contained in blackberry (BB), bilberry (Bil), black currant (BC) and elderberry (EB) extracts.

Peak number	Compound	Contained in	RT [min]	Specific fragments *m/z*			
1	cyanidin-3-*O*-sambubioside-5-*O*-glucoside	EB	6.90	743	581	449	287
2	delphinidin-3-*O*-galactoside	Bil	7.34	465	303	-	-
3	delphinidin-3-*O*-glucoside	BC	7.75	465	303	-	-
		Bil	7.90				
4	delphinidin-3-*O*-rutinoside	BC	8.40	611	465	303	-
5	cyanidin-3-*O*-galactoside	Bil	8.48	449	287	-	-
6	delphinidin-3-*O*-arabinoside	Bil	8.71	435	303		
7	cyanidin-3-*O*-glucoside	BC	8.94	449	287	-	-
		BB	9.07				
		Bil	9.12				
8	cyanidin-3-*O*-sambubioside	EB	9.03	581	449	287	-
9	petunidin-3-*O*-galactoside	Bil	9.50	479	317	-	-
10	cyanidin-3-*O*-rutinoside	BC	9.63	595	287	-	-
		BB	9.78				
11	cyanidin-3-*O*-arabinoside	Bil	9.79	419	287	-	-
12	petunidin-3-*O*-glucoside	Bil	10.01	479	317	-	-
13	petunidin-3-*O*-rutinoside	BC	10.39	625	317	-	-
14	peonidin-3-*O*-galactoside	Bil	10.50	463	301	-	-
15	petunidin-3-*O*-arabinoside	Bil	10.73	449	317	-	-
16	peonidin-3-*O*-glucoside	Bil	11.05	463	301	-	-
17	malvidin-3-*O*-galactoside	Bil	11.17	493	331	-	-
18	peonidin-3-*O*-rutinoside	BC	11.44	609	301	-	-
19	malvidin-3-*O*-glucoside	Bil	11.62	493	331	-	-
20	cyanidin-3-*O*-xyloside	BB	11.87	519	287	-	-
21	cyanidin-3-*O*-(6"-*O*-malonyl)-glucoside	BB	12.10	535	287	-	-
22	malvidin-3-*O*-arabinoside	Bil	12.34	463	331	-	-
23	cyanidin-3-*O*-(6"-*O*-dioxalyl)-glucoside	BB	12.85	593	287	-	-

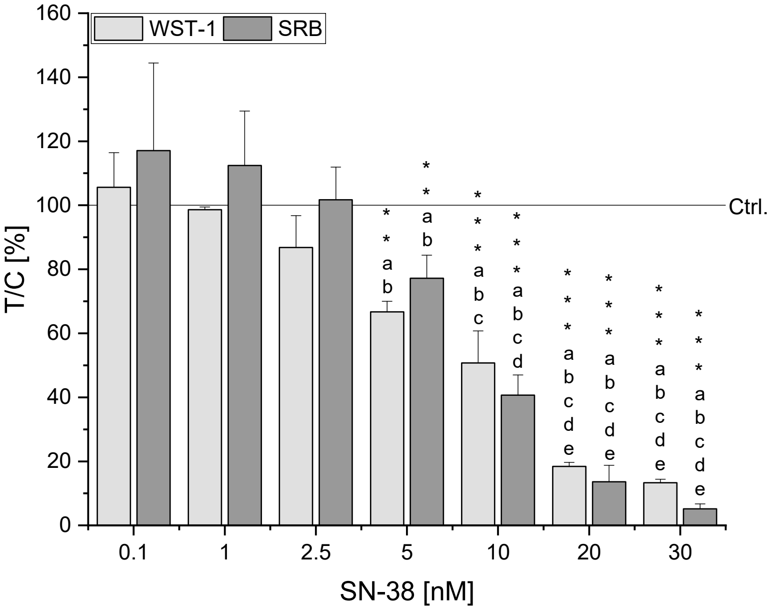

Figure S3: Cytotoxic properties of different concentrations of SN-38 assessed by the coupled WST-1 and SRB assay. CT26 cells were incubated for 72 h with SN-38. Data presented are the means + SD of at least three independent replicates measured in triplicates expressed as T/C in %. Statistical differences were calculated with one-way ANOVA via post hoc Bonferroni test (p<0.05, a-f; e.g., the letter a represents significance compared to the lowest concentration of 0.1 µM). Significances to the solvent control were calculated with one sample Student's *t*-tests (** p<0.01, *** <0.001).

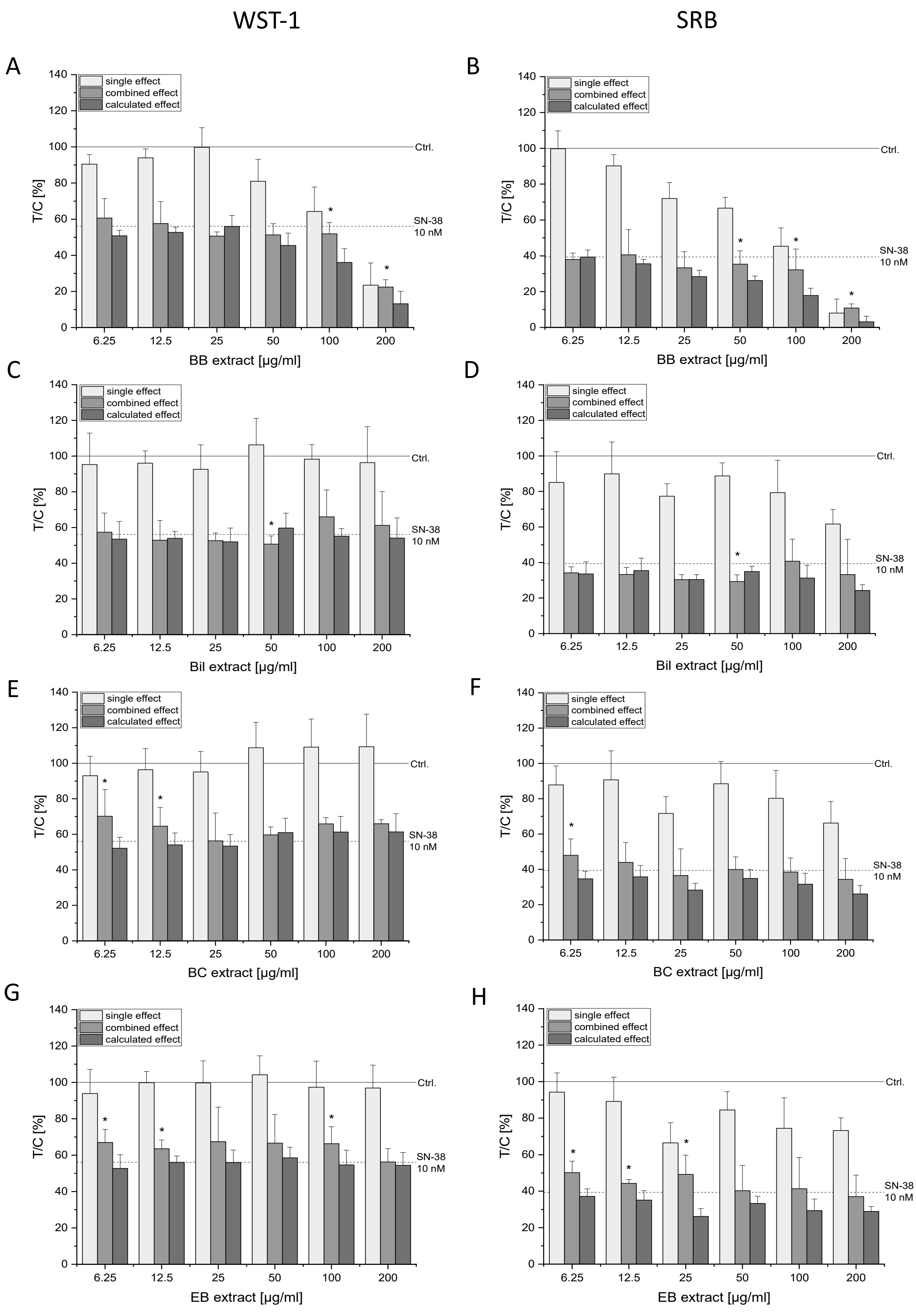

Figure S4: Independent joint action calculation from cytotoxic effects of blackberry (BB; **A**, **B**), bilberry (Bil; **B**, **C**), black currant (BC; **E**, **F**) and elderberry (EB; **G**, **H**) extracts and SN-38 (10 nM, dashed line) assessed with the WST-1 (**left**) and SRB (**right**) assay. CT26 cells were incubated for 72 hours with the respective substance. Results presented are the means + SD of the single extract effects (light grey bars), SN-38 single effect (dashed line, y=56.1 % for WST-1, y=39.4 % for SRB) and measured combined effects (middle grey bars) of at least three independent biological replicates measured in technical triplicates relative to the solvent control (Ctrl., 0.6 % DMSO, straight line, y=100 %). Dark grey bars represent the calculated combined effects of extracts and SN-38. Statistical differences between measured and calculated combined effect were evaluated with two sample Student's *t*-tests (* $p<0.05$).

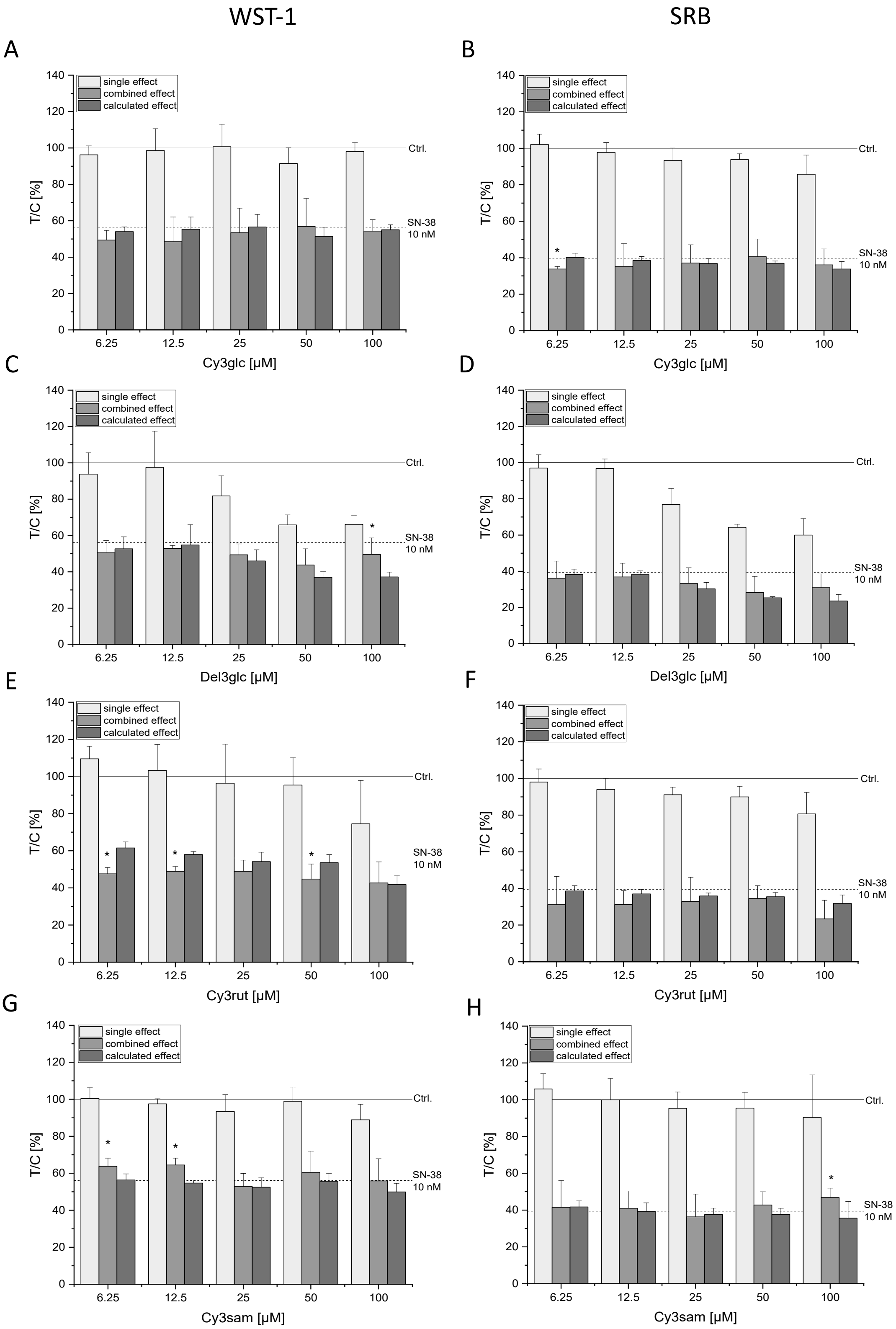

Figure S5: Independent joint action calculation from cytotoxic effects of Cy3glc (**A**, **B**), Del3glc (**B**, **C**), Cy3rut (**E**, **F**) and Cy3sam (**G**, **H**) and SN-38 (10 nM, dashed line) assessed with the WST-1 (**left**) and SRB (**right**) assay. CT26 cells were incubated for 72 hours with the respective substance. Results presented are the means + SD of the single anthocyanin effects (light grey bars), SN-38 single effect (dashed line, y=56.1 % for WST-1, y=39.4 % for SRB) and measured combined effects (middle grey bars) of at least three independent biological replicates measured in technical triplicates relative to the solvent control (0.6 % DMSO, straight line, y=100 %). Dark grey bars represent the calculated combined effects of anthocyanins and SN-38. Statistical differences between measured and calculated combined effect were evaluated with two sample Student's *t*-tests (* $p<0.05$).

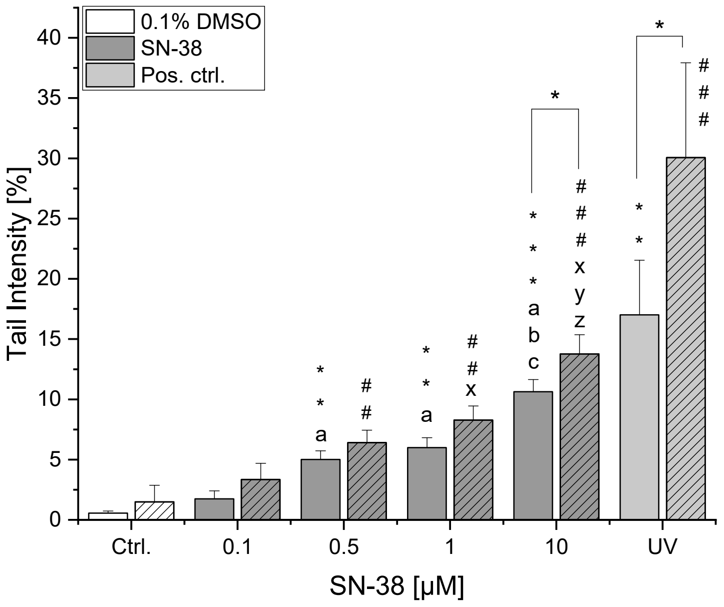

Figure S6: DNA-damaging properties of different concentrations of SN-38 assessed by the comet assay. CT26 cells were incubated for 1 h with SN38 and 1 min UV-B irradiation served as positive control. Striped bars indicate samples additionally treated with FPG enzyme. Data presented are the means + SD of at least three independent replicates expressed as tail intensity in %. Statistical differences among the tested concentrations were calculated with one-way ANOVA via post hoc Bonferroni test ($p<0.05$, a-c without FPG, x-z with FPG). Significances to the respective solvent control were calculated with two sample Student's *t*-tests (** or ## $p<0.01$, *** or ### <0.001, * without FPG, # with FPG).
